# Influence of restorative material translucency on the chameleon effect

**DOI:** 10.1038/s41598-022-12983-y

**Published:** 2022-05-25

**Authors:** Tanaporn Vattanaseangsiri, Areeyabhorn Khawpongampai, Pornpitcha Sittipholvanichkul, Nawaporn Jittapiromsak, Sumana Posritong, Kornchanok Wayakanon

**Affiliations:** 1 Department of Oral Biology, Naresuan University, Phitsanulok, Thailand; 2 Department of Restorative Dentistry, Naresuan University, Phitsanulok, Thailand; 3 Department of Medical Services, Ministry of Public Health, Nonthaburi, Thailand

**Keywords:** Biophysics, Biotechnology

## Abstract

Blending of artificial restoration materials to the natural tooth is challenging. Beyond just color, optical properties, particularly translucency, substantially influence the final appearance. The more chameleon effect that the restorative materials exhibit, the more natural looking restorations. The purpose of this study is to investigate the influence of restorative material translucency on the chameleon effect. Five types of resin composite in three different shades as well as one shade of conventional glass ionomer cement were fabricated into disks. To analyze the chameleon effect, glass ceramic blocks were milled to create four wells in each block. The restorative materials were filled into the wells. The color was measured with CIE L*a*b* every 6 months. Statistical analysis was conducted using Two-Way Repeated Measures ANOVA. The material with the highest translucency was flowable resin composite. The high translucency materials exhibited an immediate chameleon effect, as did the bulk-fill resin composites, which are low translucency. Both high and low translucency materials exhibited a delayed chameleon effect for 3 years, except for the bulk fill resin composites. The translucency of the restorative materials had a 68% positive correlation with their chameleon effect. The age of the restoration is one important factor influencing the color blending.

## Introduction

When receiving a tooth-colored restoration, all patients desire a color that blends as harmoniously as possible with the existing portion of the tooth to maintain both the natural look of the tooth as well as the effectiveness of its functions: masticating, clear speech, and normal face shape^1^. Anterior dental fillings are a delicate procedure that requires skillful use of the artificial materials to esthetically mimic the remaining natural tooth structure. Careful selection of materials is important for both the success of the restoration and for the patient’s satisfaction. Resin composite is the most frequently used tooth-colored filling material because of its excellent optical and mechanical properties^2,3^.

Resin composites are resin-based materials containing several substances that intermingle without actually interacting chemically. The physical, mechanical, and esthetic properties of resin composite result from its various components^2^. Bisphenol A-glycidyl methacrylate (Bis-GMA) and Urethane dimethacrylate (UDMA), along with the diluent monomers triethylene glycol dimethacrylate (TEGDMA) and 2-hydroxyethyl methacrylate (HEMA), are commonly used to create the desired viscosity in polymerizable materials^4,5^. Camphoroquinone and the primary amine dimethylamine ethyl methacrylate (DMAEMA) are general photosensitizers which are added to create photopolymerizable resin composite^6^. The majority of resin composites in the market are made using nanotechnology to produce nanofilled or nanohybrid resin composites, which gives them similar mechanical properties. The filler particles are important to improve the physical and mechanical properties of the materials by reducing the thermal expansion coefficient and polymerization shrinkage, providing radiopacity, and improving the handling and esthetics of materials^7^. The polymerization shrinkage is the significant problem for long time. The shrinkage of the material has been continuously improved over time and at present is only 1.72–2.13%^8^. However, the optical properties of a composite are the central concern when trying to determine which composite will result in the most natural-looking restoration.

Matching the best resin composite to the specific characteristics of the patient’s remaining tooth structure can be challenging, and color matching is the first important step. Value, chroma, and hue are three fundamental dimensions of color that are crucial to consider in routine practice. However, the appearance of a tooth is a complex phenomenon because of its interactions with light, which include absorption, transmission, reflection, refraction, and scattering. Translucency refers to the relative amount of light that passes through and exits an object after undergoing mainly absorption and scattering^9^. Translucence, opalescence, and fluorescence are the three optical properties most important in dentistry, and among these three, translucence has the greatest influence on the esthetics of a dental restoration^10,11^. For example, translucence affects the “color blending” on the tooth, the masking ability of the composite, and the penetration of light curing^12^.

Color blending, or color assimilation, refers to the perception that an existing color difference between the restorative material and the remaining tooth structure is perceived as smaller when the two colors are physically adjacent to each other at the restoration site than when they are viewed separately at some distance from each other. This phenomenon is also commonly known as the chameleon effect^13,14^. A variety of factors are known to affect the chameleon effect: the type and shade of the resin composite^15^, the amount of color difference between the tooth and the restoration^13^, and the size^13^ and thickness of the restoration^16^. When the resin composite is filled into the cavity, the colors and optical properties of the resin composite, the resin-covered tooth structure underneath, and the remaining exposed tooth structure all interact, and together they affect light interaction and lead to the chameleon effect^15^.

Resin composites are the most frequently used dental restorative materials, since they are able to render restorations imperceptible when the appropriate translucency is chosen. The translucency of the material allows the underlying and adjacent remaining tooth structure to show through the restoration^17^ and makes it look harmonious. However, a wide variety of resin composites are currently available, and their translucency varies. Rather than using a specific, quantified indicator of translucency level, the degree of a resin’s translucency is indicated broadly by three general resin composite types: “enamel”, “body/universal”, or “dentin”^18^. Enamel type has the highest translucency and dentin type has the lowest. These three types are used to describe only conventional resin composites. However, flowable resin composites, bulk-fill resin composites, and glass ionomer cements are also commonly used clinically, and how their translucency compares to the conventional resins is not well understood. Unfortunately, with no current standardized system for numerically quantifying the optical properties of resin composite, particularly the translucency property, misselection of resin composite and disappointing restorative results frequently occur.

The purpose of this study is to investigate how the translucency of various resin composite types influences their chameleon effect. A better understanding of the blending capabilities of each type of resin composite can assist clinicians in making the most effective selections.

## Materials and methods

### Investigating the translucency parameter of different types of resin composites and glass ionomer cement

This study uses five types of resin composite (enamel, body, dentin, flowable, and bulk-fill) in three different shades (A1, A2, and A3) as well as one shade (A2) of conventional glass ionomer cement. These sixteen experimental groups are shown with their codes and product information in Table 1.

**Table 1:** The experimental groups in this study.

	Enamel^1^	Body^1^	Dentin^1^	Flowable^2^	Bulk-fill^3^	GI^4^
A1	A1E	A1B	A1D	A1F	A1BF	
A2	A2E	A2B	A2D	A2F	A2BF	GI
A3	A3E	A3B	A3D	A3F	A3BF	

Products: ^1^Filtek™ Z350 XT; 3M ESPE, St. Paul, USA.

^2^Filtek™ Supreme Ultra Flowable Restorative; 3M ESPE, St. Paul, USA.

^3^Filtek™ One Bulk Fill Restorative; 3M ESPE, St. Paul, USA.

^4^3M™ Ketac™ Universal Aplicap™ Glass Ionomer Restorative; 3M ESPE, St. Paul, USA.

Resin composite and glass ionomer cement samples were formed in the shape of disks with a diameter of 10-mm and a thickness of 0.5-mm using acrylic molds. There were five identical samples of each of the 16 experimental groups (N = 80). A glass slide was placed on top of the samples to prevent an oxygen inhibition layer from forming during 40 s of blue curing light 1,250 mW/cm^2^ in a spectrum of wavelength between 440 and 460 nm (Mini LED™ Standard; ACTEON, Mount Laurel, New Jersey, USA). The translucency of each sample was measured by finding its Translucency Parameter (TP). In order to do that, the color of samples when placed alternately on black paper and white paper was determined in three dimensions (L*a*b*) using a spectrophotometer (VITA Easyshade® V; VITA Zahnfabrik, Bad Säckingen, Germany) according to International Commission on Illumination (CIE) protocols. The measured values were then inserted into the following formula to calculate the Translucency Parameter^19,20^:

$${\text{TP}} = \left[ {\left( {{\text{L}}_{{\text{b}}} - {\text{L}}_{{\text{w}}} } \right)^{{2}} + \left( {{\text{a}}_{{\text{b}}} - {\text{a}}_{{\text{w}}} } \right)^{{2}} + \left( {{\text{b}}_{{\text{b}}} - {\text{b}}_{{\text{w}}} } \right)^{{2}} } \right]^{{{1}/{2}}}$$

L_b_ = Lightness of the sample on black paper, L_w_ = Lightness of the sample on white paper, a_b_ = Redness of the sample on black paper, a_w_ = Redness of the sample on white paper, b_b_ = Yellowness of the sample on black paper, b_w_ = Yellowness of the sample on white paper.

### Investigating the chameleon effect of different types of resin composite and glass ionomer cement aged for 5 years

**Figure 1:**
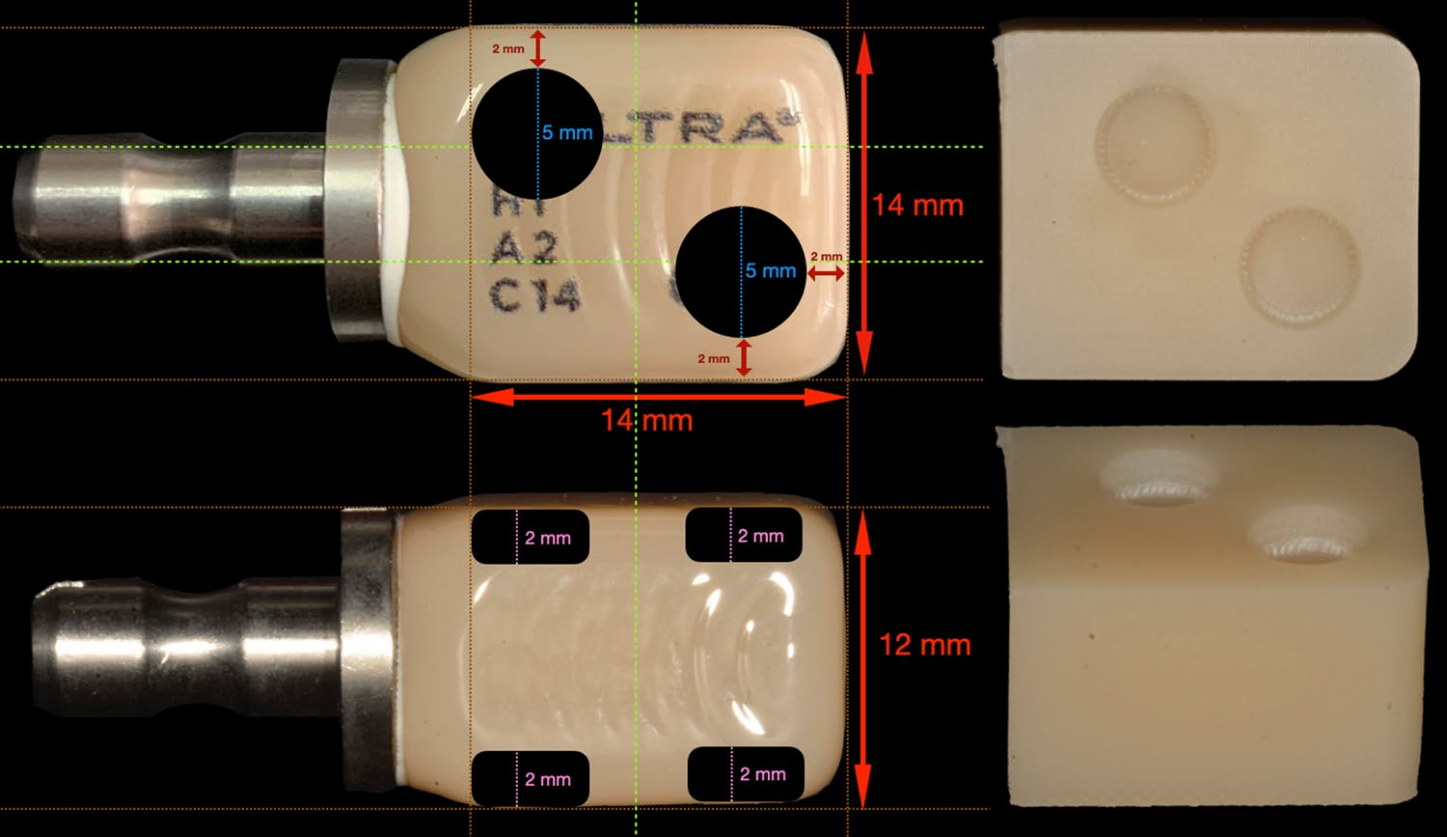
Design for creating 4 wells on the top and bottom surfaces of glass ceramic blocks.

Thirty-two glass ceramic blocks (Celtra Duo®; Dentsply Sirona; Charlotte, North Carolina, USA) in shade A2 were designed with Computer-Aided Design (CAD) software (Powershape 2020, Autodesk Inc., California, United States) and milled with a milling unit (Coritec 250i, imes-icore GmbH, Eiterfeld, Germany) to create four wells (two on the top, two on the bottom) with a depth of 2-mm and a diameter of 6-mm in each block (Fig. 1). The milled blocks were sintered at 840 °C for 30 min, and then divided into 16 groups (n = 2) as shown in Table 1.

The inner surfaces of each well were prepared by applying 4.9% hydrofluoric acid (hydrofluoric acid; Porcelain Etch®, Ultradent, South Jordan, Utah, USA) for 20 s, rinsing with water for 30 s, and then drying with air blow. Then silane coupling agent (Ultradent, South Jordan, Utah, USA) was applied for 1 min, followed air blowing (no rinsing)^21,22^. Next, dental adhesive agent (Single Bond® Universal Adhesive; 3M ESPE, St. Paul, Minnesota, USA) was applied to the well surfaces. A new microbrush was used to remove the excess bonding agent, leaving a thin film, followed by air blowing and finally light curing for 20 s. With the wells thus prepared, each resin composite material in Table 1 was placed in a total of 8 channels (using 2 ceramic blocks) with the bulk technique. A glass slide was placed on top of the filled materials, followed by light curing for 40 s. In the case of the glass ionomer cement, the surfaces of the ceramic channels were prepared in the same manner as for the resin composite, except that neither the silane nor the dental adhesive reagent was applied. Also, the glass ionomer cement, covered by the glass slide, was allowed to cure for 7 min. Table 2 describes the composition of the materials used.

**Table 2:** Components of the materials used in this study.

Material	Composition
Filtek™ Z350 XT	Bis-GMA, UDMA, TEGDMA, Bis-EMA and fillers 78.5% by weight (63.3% by volume): a combination of non-agglomerated/non-aggregated 20 nm silica filler, non-agglomerated/non-aggregated 4 to 11 nm zirconia filler, and aggregated zirconia/silica cluster filler (comprised of 20 nm silica and 4 to 11 nm zirconia particles)
Filtek™ Supreme Ultra Flowable Restorative	BisGMA, TEGDMA, Procrylat resins and fillers 65% by weight (46% by volume): a combination of ytterbium trifluoride filler 0.1 to 5.0 μm, a non-agglomerated/non-aggregated surface-modified 20 nm silica filler, a non-agglomerated/ non-aggregated surface modified 75 nm silica filler, and a surface-modified aggregated zirconia/silica cluster filler 0.6 to 10 μm (comprised of 20 nm silica and 4 to 11 nm zirconia particles)
Filtek™ One Bulk Fill Restorative	AUDMA, UDMA and 1, 12-dodecane-DMA, and fillers 76.5% by weight (58.4% by volume): a combination of a non-agglomerated/non-aggregated 20 nm silica filler, a non-agglomerated/ non-aggregated 4 to 11 nm zirconia filler, an aggregated zirconia/silica cluster filler (comprised of 20 nm silica and 4 to 11 nm zirconia particles) and agglomerate 100 nm particles ytterbium trifluoride filler
Ketac™ Universal Aplicap™ Glass Ionomer Restorative	Powder: Oxide glass > 95% by weight Liquid: Water, copolymer of acrylic acid-maleic acid, tartaric acid and benzoic acid
Celtra Duo® ceramic block	Silicon dioxide (58% by weight), phosphorus pentoxide (5% by weight), alumina (1.9% by weight), lithium oxide (18.5% by weight), zirconium dioxide (10.1% by weight), terbium oxide (1% by weight), ceria (2% by weight) and zirconium dioxide diluted completely in glass matrix (10% by weight)
Porcelain Etch®	4.9% hydrofluoric acid
Silane	3-methacryloxypropyltrimethoxysilane, ethanol
Single Bond Universal Adhesive	10-MDP Phosphate Monomer, HEMA, dimethacrylate resin, Vitrebond™ Copolymer, filler, ethanol, water, initiators, silane

After the restorative materials were filled into the ceramic wells, all specimens were soaked in distilled water at room temperature for 24 h. After that, the samples were briefly daubed with a paper towel to remove any beads of water, and while they remained in a moist condition, the spectrophotometer (VITA Easyshade® V; VITA Zahnfabrik, Bad Säckingen, Germany) was used to measure the color properties (CIE L*a*b*) of the restorative materials, along with those of the adjacent ceramic material. This machine has an internal ceramic calibrating device which faces the tip of the machine for calibration once the machine is turned on. These initial measurements were recorded as Day 0. The specimens were then stored in distilled water at 37 °C and the color properties were again measured after 7, 14, and 30 days. Next, all specimens were thermocycled (SDC20 HWB332R, Yamatake Honeywell, Japan) in water between 5 °C and 55 °C with a 15 s dwelling time. Under these conditions, 5,000 cycles represent approximately 6 months of in vivo temperature transition. The thermocycling continued for an in vivo equivalent of 5 years, and at each 6 months in vivo equivalent, the color properties of the restorative materials and adjacent ceramic material were measured and recorded.

∆E between any two samples (ceramic and each restorative material) was calculated from their respective color parameters (L*a*b*) using the following formula^23,24^:

$$\triangle{{\text{E}}=\left[{\left({{\text{L}}_{{{\text{ceramic}}}} - {\text{L}}_{{{\text{restorative}}\;{\text{material}}}} } \right)^{{2}} + \left( {{\text{a}}_{{{\text{ceramic}}}} - {\text{a}}_{{{\text{restorative}}\;{\text{material}}}} } \right)^{{2}} + \left( {{\text{b}}_{{{\text{ceramic}}}} - {\text{b}}_{{{\text{restorative}}\;{\text{material}}}}}\right)^{{2}}}\right]^{{{1}/{2}}}}$$

L_ceramic_ = Lightness of ceramic, L_restorative material_ = Lightness of restorative material, a_ceramic_ = Redness of ceramic, a_restorative material_ = Redness of restorative material, b_ceramic_ = Yellowness of ceramic, b_restorative material_ = Yellowness of restorative material.

### Data analysis

The average color property (CIE L*a*b*) of each material was calculated from all five samples in each group, and each sample was measured three times by spectrophotometer. After the mean translucency and standard deviation as well as the mean ∆E and standard deviation were calculated in a wide variety of sample comparisons, these figures were analyzed for statistically significant differences using One-Way ANOVA in SPSS statistical software (SPSS 26.0, SPSS Inc., Chicago, IL, USA). The significance level was set at 0.05. In the statistical analysis of the color parameters, the means and standard deviations were analyzed using Two-Way ANOVA with repeated measures at the significance level of 0.05. The relationship between translucency and ∆E was analyzed using Regression Analysis at the significance level of 0.05.

## Results

### Investigating the translucency parameter of different types of restorative materials

**Figure 2:**
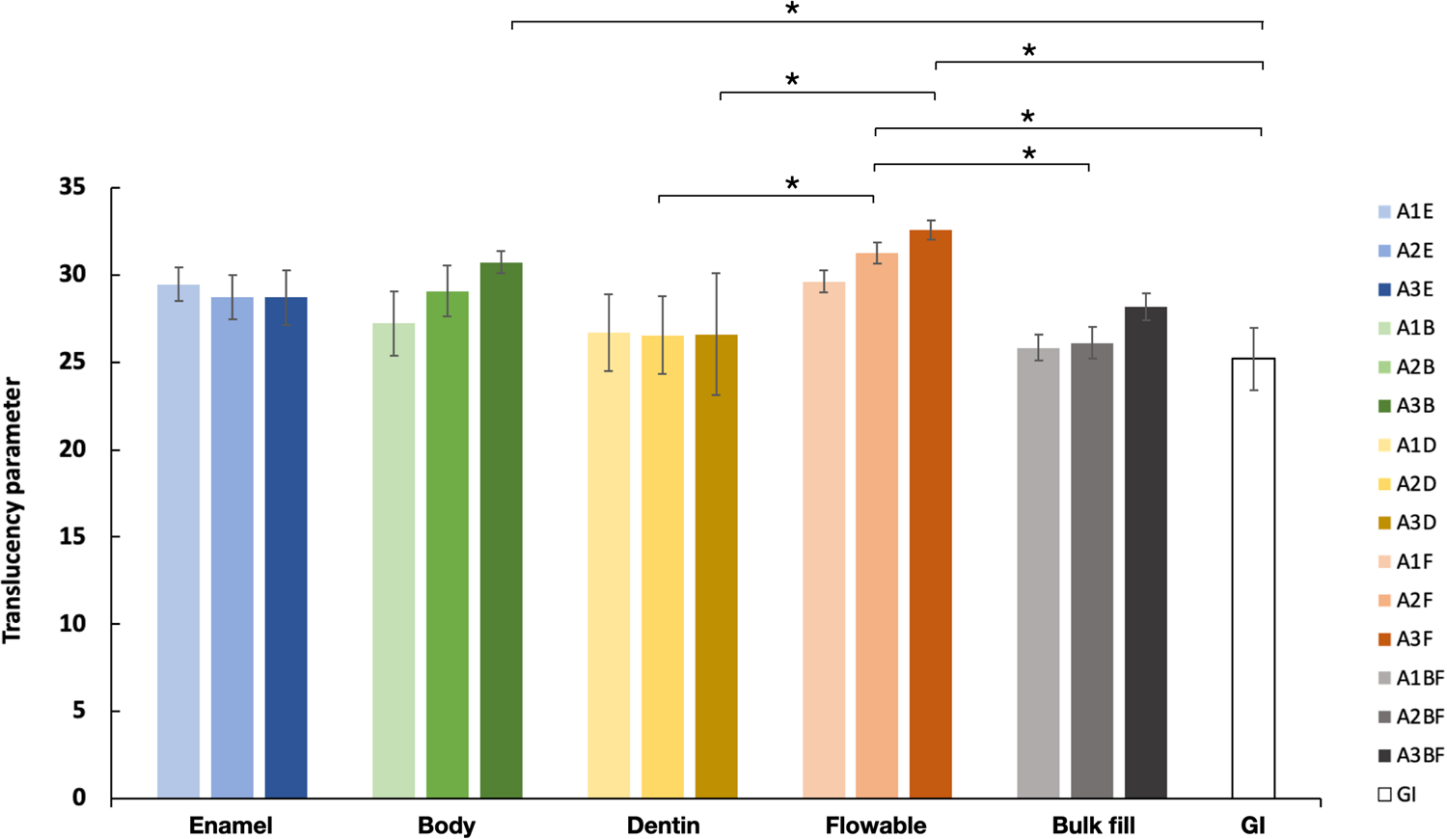
The translucency parameter of each shade of different types of restorative materials. *Indicates significant difference (*p* < 0.05).

The translucency parameters of all the restorative material samples were calculated using CIE L*a*b*, and the results are shown in Fig. 2. Within each type of restorative material, there was no statistical difference in the translucency parameter of the three shades (A1, A2, and A3) in any of the materials (*p* > 0.05). Searching for translucency differences between material types, all materials fell into one of two groups: a high translucency group (enamel, body, and flowable resin composites) and a low translucency group (dentin, bulk-fill resin composites and GI). Other various observations include the following. Within the same shade, the dentin and bulk-fill resin composites had similar lower translucency parameters compared to others. The flowable resin composite had the highest translucency parameter in every shade. In the A2 shade, the flowable resin had a significantly higher translucency parameter compared to the same shade of the dentin (*p* = 0.05), bulk-fill (*p* = 0.024), and glass ionomer (*p* = 0.004). A similar situation was found in the A3 shade, in which the flowable resin had a significantly higher translucency parameter than the dentin (*p* = 0.005). The glass ionomer cement had the lowest translucency parameter of all the materials, significantly lower than the A2 (same shade) (*p* = 0.004) and A3 (*p* < 0.001) of the flowable resin composite and the A3 of the body resin composite (*p* = 0.012). The pattern that emerges from these observations is that the type of material can significantly influence the translucency parameter while the shade has no significant influence.

### Investigating changes in color properties of different types of restorative materials across 5 years

After the three shades (A1, A2 and A3) of five types (enamel, body, dentin, flowable and bulk-fill) of resin composite and A2 conventional glass ionomer cement were placed in the wells of the A2 glass ceramic blocks and underwent temperature transitions for the equivalent of 5 years, there were color alterations in all the materials.

Changes in the lightness of the restorative materials and the ceramic across 5 years are shown in Table 3. The lightness of the ceramic significantly decreased in the first 3 years (*p* < 0.05). Thereafter, the ceramic lightness recovered and was again comparable to the initial measurement (*p* = 0.775). All the restorative materials also decreased in lightness as time passed, compared to their initial measurement. Even though on Day 0 the A1 and A2 shades of enamel, dentin, and bulk-fill resin composite started with lightness that was significantly higher than that of ceramic, after one year their lightness was no longer significantly different from the ceramic. Thereafter, enamel and bulk-fill resin composite retained a lightness relatively comparable to ceramic until the five year mark while the dentin resin composite lightness remained comparable to ceramic for only 4 years.

**Table 3:** Changes in lightness of the restorative materials and the ceramic across 5 years.

Time						
Material	L*(0 day)	L*(1 year)	L*(2 years)	L*(3 years)	L*(4 years)	L*(5 years)
Ceramic	72.72 ± 1.35^aA^	72.05 ± 1.60^beA^	71.71 ± 1.64^ceA^	71.61 ± 1.60^bcdA^	72.19 ± 1.74^eA^	72.07 ± 1.88^abeA^
A1E	77.66 ± 2.05^aB^	72.03 ± 0.96^bA^	71.75 ± 0.90^bA^	71.58 ± 0.84^bA^	71.85 ± 1.04^bA^	71.41 ± 0.88^bA^
A2E	75.85 ± 1.50^aB^	71.03 ± 0.95^bA^	70.59 ± 1.05^bA^	71.28 ± 2.35^bA^	70.56 ± 1.54^bA^	70.51 ± 1.52^bA^
A3E	74.10 ± 1.49^aA^	69.21 ± 1.23^cA^	68.30 ± 1.76^bcB^	67.99 ± 3.56^bcA^	67.70 ± 3.62^bcA^	65.45 ± 5.80^bB^
A1B	76.79 ± 3.9^aB^	69.8 ± 1.69^cA^	67.50 ± 2.78^ dB^	64.58 ± 3.80^eB^	61.86 ± 4.50^fB^	63.03 ± 7.96^befB^
A2B	76.48 ± 3.21^aB^	70.66 ± 3.77^cA^	70.34 ± 2.62^bcA^	69.45 ± 3.98^bcA^	67.44 ± 6.48^dA^	65.55 ± 7.20^ dB^
A3B	75.74 ± 3.06^aB^	67.94 ± 2.32^cB^	67.81 ± 2.02^bcB^	66.85 ± 3.15^bcB^	64.40 ± 3.70^ dB^	64.40 ± 4.82^bcdB^
A1D	78.50 ± 2.53^aB^	70.80 ± 4.89^cA^	70.79 ± 4.34^bcA^	69.41 ± 6.23^bcA^	67.10 ± 5.72^dA^	64.86 ± 7.38^ dB^
A2D	78.65 ± 1.86^aB^	74.49 ± 1.53^cA^	73.83 ± 2.41^bceA^	73.28 ± 1.45^bcA^	71.35 ± 2.66^deA^	70.19 ± 3.79^dA^
A3D	74.73 ± 2.71^aA^	66.91 ± 3.45^cB^	64.41 ± 4.17^ dB^	63.13 ± 6.21^bdB^	58.95 ± 8.26^eB^	56.86 ± 9.67^eB^
A1F	75.40 ± 3.50^aB^	69.56 ± 2.59^cA^	68.65 ± 2.80^bcB^	68.98 ± 2.01^bcA^	68.45 ± 1.98^bcA^	64.86 ± 3.31^ dB^
A2F	71.73 ± 1.27^aA^	66.68 ± 1.75^bcB^	66.03 ± 1.93^bcB^	65.06 ± 2.56^bcB^	66.61 ± 2.46^cB^	63.95 ± 2.61^bB^
A3F	71.79 ± 1.77^aA^	66.64 ± 0.47^bcB^	66.21 ± 1.05^bcB^	66.38 ± 0.99^bcB^	67.29 ± 1.76^cA^	64.68 ± 2.01^bB^
A1BF	75.90 ± 0.81^aB^	73.64 ± 1.87^bA^	73.61 ± 2.19^bA^	74.10 ± 3.17^abA^	73.81 ± 3.05^abA^	72.76 ± 3.98^abA^
A2BF	74.35 ± 1.93^aA^	70.88 ± 3.28^bcA^	70.51 ± 2.38^bcA^	70.13 ± 5.29^bA^	71.50 ± 3.59^abA^	67.36 ± 5.26^cA^
A3BF	71.29 ± 1.69^aA^	67.41 ± 3.77^cB^	67.31 ± 3.88^bcB^	66.71 ± 5.35^bcB^	65.78 ± 6.76^bcdB^	63.58 ± 8.97^ dB^
GI	70.16 ± 4.09^aA^	68.64 ± 3.38^aB^	63.31 ± 3.71^cB^	60.35 ± 9.78^ dB^	56.88 ± 13.21^bB^	57.43 ± 14.26^bB^

Lower case letters indicate significant difference within the row (*p* < 0.05).

Upper case letters indicate significant difference within the column (*p* < 0.05).

Changes in the redness of the restorative materials and the ceramic are shown in Table 4. The redness of the ceramic did not change significantly throughout 5 years. The initial redness of the ceramic was significantly lower (more greenness) than all the types of restorative materials, even those having the same shade. All the restorative materials except the dentin resin composite also decreased in redness when compared to their initial measurement. The dentin resin composite changed in the opposite direction, increasing in redness.

**Table 4:** Changes in redness of the restorative materials and the ceramic across 5 years.

Time						
Material	a*(0 day)	a*(1 year)	a*(2 years)	a*(3 years)	a*(4 years)	a*(5 years)
Ceramic	− 1.40 ± 0.23^acA^	− 1.41 ± 0.26^abA^	− 1.34 ± 0.26^abA^	− 1.40 ± 0.28^abA^	− 1.18 ± 0.31^cA^	− 1.24 ± 0.59^acA^
A1E	− 1.44 ± 0.37^aB^	− 1.48 ± 0.45^aB^	− 1.40 ± 0.47^aB^	− 1.48 ± 0.49^aB^	− 1.25 ± 0.50^aB^	− 1.31 ± 0.71^aB^
A2E	− 0.65 ± 0.26^aB^	− 1.26 ± 0.14^aA^	− 1.25 ± 0.14^aA^	− 1.19 ± 0.36^aA^	− 1.13 ± 0.31^aA^	− 1.21 ± 0.38^aA^
A3E	0.39 ± 0.26^aB^	− 0.70 ± 0.51^bA^	− 0.90 ± 1.14^bA^	− 1.15 ± 1.35^bA^	− 1.16 ± 1.40^bA^	− 1.15 ± 1.70^bA^
A1B	− 0.56 ± 0.18^aB^	− 3.48 ± 0.77^bB^	− 4.03 ± 1.33^cB^	− 5.41 ± 1.68^ dB^	− 4.58 ± 1.79^ceB^	− 3.79 ± 2.02^bcB^
A2B	0.41 ± 0.31^aB^	− 0.51 ± 0.48^cB^	− 1.06 ± 1.17^bdA^	− 1.63 ± 1.69^dA^	− 0.95 ± 1.37^bcA^	− 0.93 ± 1.09^bcdA^
A3B	0.31 ± 0.67^abB^	0.50 ± 0.69^aB^	0.38 ± 1.08^aB^	− 0.46 ± 1.65^bA^	− 0.19 ± 1.27^abA^	− 0.18 ± 1.32^abA^
A1D	0.44 ± 0.21^aB^	1.39 ± 0.73^cdB^	2.11 ± 0.45^bB^	0.74 ± 2.14^acB^	1.76 ± 1.28^bdB^	1.49 ± 1.86^abcB^
A2D	0.33 ± 0.17^aB^	0.93 ± 0.55^adB^	1.45 ± 0.49^cB^	1.75 ± 0.79^bcdB^	2.13 ± 0.98^bB^	2.21 ± 0.85^bcB^
A3D	0.90 ± 0.08^abB^	0.69 ± 1.89^abB^	1.01 ± 1.68^abB^	0.34 ± 2.57^bB^	1.08 ± 1.67^aB^	1.44 ± 1.58^aB^
A1F	− 1.88 ± 0.23^aB^	− 2.75 ± 0.42^bcB^	− 3.04 ± 0.57^bcB^	− 3.51 ± 0.77^cB^	− 2.83 ± 0.47^abB^	− 2.85 ± 0.71^abcB^
A2F	− 0.94 ± 0.30^aB^	− 1.50 ± 1.03^adA^	− 2.00 ± 0.43^cA^	− 2.88 ± 0.39^bB^	− 1.44 ± 0.64^acA^	− 2.13 ± 0.62^bcdA^
A3F	− 0.18 ± 0.88^abcdB^	0.29 ± 0.39^bcB^	0.35 ± 0.35^abB^	− 0.18 ± 0.55^abcdA^	− 0.66 ± 0.76^dA^	− 0.05 ± 0.52^abcdA^
A1BF	− 2.20 ± 0.27^aB^	− 2.68 ± 0.26^aB^	− 2.83 ± 0.39^aB^	− 3.11 ± 0.82^aB^	− 2.91 ± 0.73^aB^	− 2.94 ± 0.67^aB^
A2BF	− 1.49 ± 0.14^aA^	− 2.03 ± 0.19^abA^	− 2.25 ± 0.61^bB^	− 2.78 ± 1.17^bB^	− 2.39 ± 1.48^abB^	− 2.75 ± 1.21^bB^
A3BF	− 0.29 ± 0.29^aB^	− 0.98 ± 0.59^cA^	− 1.13 ± 1.19^bcA^	− 2.00 ± 2.19^dA^	− 1.74 ± 1.93^dA^	− 1.79 ± 2.15^bcdA^
GI	5.46 ± 0.76^aB^	− 2.01 ± 1.15^cA^	− 2.96 ± 0.76^bB^	− 3.09 ± 1.97^bB^	− 3.29 ± 1.25^bB^	− 3.60 ± 1.86^bB^

Lower case letters indicate significant difference within the row (*p* < 0.05).

Upper case letters indicate significant difference within the column (*p* < 0.05).

Changes in the yellowness of the restorative materials and the ceramic are shown in Table 5. The yellowness of the ceramic significantly increased at the 2 year point (*p* = 0.001) and continued to increase thereafter. The yellowness of the restorative materials fluctuated during the 5 years. However, the majority of the materials in shade A1 increased in yellowness while the A2 and A3 shades decreased, except for the yellowness of all three shades of flowable resin composite, which increased. Compared to the ceramic, the majority of the restorative materials had higher yellowness initially. After 2 years, the yellowness of the ceramic and all restorative materials were not significantly different. After that, the majority of the restorative materials once again had higher yellowness than the ceramic.

**Table 5:** Changes in yellowness of the restorative materials and the ceramic across 5 years.

Time						
Material	b*(0 day)	b*(1 year)	b*(2 years)	b*(3 years)	b*(4 years)	b*(5 years)
Ceramic	12.72 ± 1.05^aA^	12.97 ± 1.04^abA^	13.39 ± 1.07^cA^	13.25 ± 1.21^bcA^	14.86 ± 1.27^dA^	14.84 ± 2.47^dA^
A1E	12.58 ± 1.56^aA^	12.5 ± 2.13^bB^	12.9 ± 2.22^abA^	12.76 ± 2.17^bB^	14.38 ± 2.33^bB^	14.34 ± 3.19^bB^
A2E	15.66 ± 1.16^aB^	11.66 ± 0.47^bA^	11.74 ± 0.3^abA^	11.73 ± 0.66^bA^	12.65 ± 0.44^bA^	12.25 ± 0.47^bA^
A3E	18.46 ± 1.74^aB^	15.38 ± 0.52^cB^	16.18 ± 0.94^abcA^	16.10 ± 1.5^bcB^	17.64 ± 2.25^aB^	17.78 ± 2.46^abA^
A1B	19.30 ± 3.42^aB^	15.70 ± 1.49^bB^	17.05 ± 1.45^abA^	16.86 ± 1.16^bB^	21.01 ± 1.71^aB^	20.64 ± 2.38^aB^
A2B	21.19 ± 2.59^aB^	17.44 ± 0.82^bB^	18.06 ± 0.8^abA^	17.36 ± 0.67^bB^	20.73 ± 2.92^aB^	20.36 ± 3.02^aB^
A3B	25.74 ± 3.24^aB^	20.11 ± 1.07^cB^	21.34 ± 0.75^abcdA^	19.59 ± 1.36^bcB^	22.78 ± 1.22^ dB^	23.09 ± 1.3^adB^
A1D	30.28 ± 2.3^aB^	24.74 ± 1.82^cB^	24.96 ± 2.03^acdeA^	23.33 ± 1.58^bdB^	26.49 ± 1.65^eB^	25.84 ± 1.46^ceB^
A2D	35.53 ± 1.94^aB^	31.94 ± 1.39^cB^	32.03 ± 1.20^bcB^	28.94 ± 1.52^ dB^	32.54 ± 1.13^cB^	32.04 ± 0.81^cB^
A3D	36.28 ± 5.04^aB^	31.08 ± 2.31^cB^	31.55 ± 1.29^abcdA^	28.24 ± 4.4^ dB^	33.56 ± 0.9^bB^	32.43 ± 1.83^bcB^
A1F	14.81 ± 2.17^aceA^	13.44 ± 1.42^cA^	13.24 ± 1.87^acdA^	13.65 ± 2.22^acA^	17.95 ± 1.4^bdB^	17.31 ± 1.54^edA^
A2F	17.21 ± 2.18^aB^	18.09 ± 3.49^aB^	18.53 ± 3.73^abA^	17.83 ± 4.73^aB^	21.93 ± 2.15^bB^	19.85 ± 2.41^aB^
A3F	24.21 ± 1.91^aB^	22.13 ± 1.08^cB^	23.26 ± 1.27^abcdA^	21.10 ± 1.70^bcB^	26.75 ± 1.36^ dB^	25.39 ± 1.50^adB^
A1BF	11.36 ± 0.81^aA^	9.33 ± 0.91^bB^	9.68 ± 1.12^abA^	9.09 ± 1.05^bB^	10.56 ± 1.52^abB^	10.23 ± 1.47^abB^
A2BF	13.09 ± 1.05^aA^	10.59 ± 0.3^bB^	10.94 ± 0.6^abA^	10.46 ± 0.85^bB^	12.45 ± 0.99^aB^	11.81 ± 1.79^abA^
A3BF	16.58 ± 0.79^aB^	14.35 ± 2.18^bA^	14.96 ± 2.2^abA^	14.13 ± 2.26^bA^	15.55 ± 3.01^abA^	15.78 ± 3.1^abA^
GI	43.69 ± 2.26^aB^	35.58 ± 2.48^cB^	33.11 ± 2.58^abcdA^	28.51 ± 7.67^ dB^	32.74 ± 5.56^bB^	32.30 ± 6.47^bB^

Lower case letters indicate significant difference within the row (*p* < 0.05).

Upper case letters indicate significant difference within the column (*p* < 0.05).

### Investigating ∆E of the ceramic across 5 years

The specific color properties of the ceramic at various points in time were shown in the previous section. Table 6 shows the resulting ∆E of the ceramic across the 5 years. The ∆E of the ceramic increased continuously as time passed. At the 2 year point, the color of the ceramic was significantly different from its initial color (*p* < 0.001), and the color of the ceramic continued to change throughout the 5 years of this study.

**Table 6:** The ∆E of the ceramic across 5 years.

	Day 0	1 Year	2 Years	3 Years	4 Years	5 Years
Day 0						
1 Year	0.96 ± 0.6^A^					
2 Years	1.34 ± 0.88^aB^	0.81 ± 0.52^bA^				
3 Years	1.45 ± 0.84^aB^	0.96 ± 0.60^bA^	0.77 ± 0.45^bA^			
4 Years	2.39 ± 0.97^aC^	2.09 ± 0.66^acB^	1.73 ± 0.51^bB^	1.86 ± 0.50^bcA^		
5 Years	2.58 ± 2.18^aC^	2.26 ± 2.02^acB^	1.94 ± 2.05^bB^	2.12 ± 2.08^bcA^	0.98 ± 2.16^dA^	

Lower case letters indicate significant difference within the row (*p* < 0.05).

Upper case letters indicate significant difference within the column (*p* < 0.05).

### Investigating the ∆E between the restorative materials and the adjacent ceramic across 5 years

**Figure 3:**
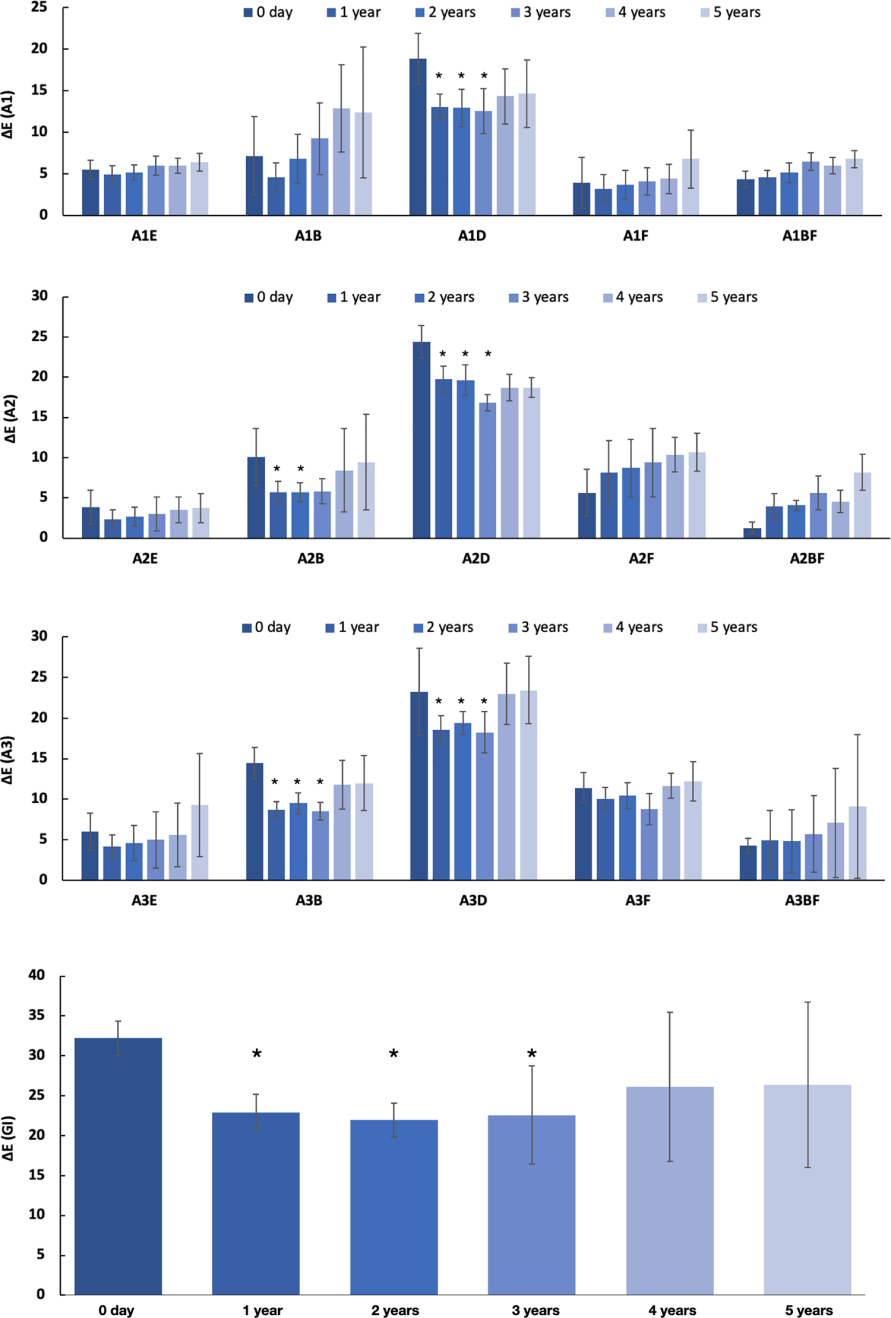
The ∆E between each restorative material and its adjacent ceramic across 5 years. *Indicates significant difference compared with Day 0 (*p* < 0.05).

It is clear that color property changes occur in all types of restorative materials as well as in the adjacent ceramic. The chameleon effect refers to differences in the perception of a substance’s color, depending on the surrounding environment. In order to investigate the chameleon effect, this study examined the ∆E between the restorative materials and the adjacent ceramic environment. The results are shown in Fig. 3.

A majority of the restorative materials and their adjacent ceramic showed a lower ∆E after 1, 2, and 3 years than their initial ∆E. Exceptions were all shades of the bulk-fill resin composite and the A2 shade of the flowable resin composite. Compared to the initial readings, the body resin composite A2 had significantly lower ∆E at the 1 year and 2 year points (*p* = 0.03 and 0.12, respectively), while A3 had significantly lower ∆E at the 1, 2, and 3 year points (*p* < 0.001, *p* = 0.02 and *p *= 0.03, respectively). The ∆E of the A1 body resin composite also decreased after 1 year, but only insignificantly (*p* = 0.357). The ∆E of all three shades of the dentin resin composite as well as the glass ionomer cement significantly decreased (*p* < 0.05) at the 1, 2, and 3 year points, compared to the initial measurement. The ∆E of the A1 and A3 flowable resin composites decreased only insignificantly (*p* > 0.05) after 1, 2, and 3 years, compared to their initial ∆E. The ∆E of the A2 flowable resin composite and all shades of the bulk-fill resin composite insignificantly increased at all the checkpoints (Years 1–5), compared with the initial ∆E.

### Correlation between translucency and the chameleon effect

Each material has its own measurable degree of translucency. On the other hand, the chameleon effect, as a concept of perception by the human eye, would be rather messy to measure directly. Instead, this study looks at the underlying definable color properties of the two materials being compared to see what might be responsible for causing the human perception. The ∆E between each restorative material and the environment (the ceramic) is used here to represent the differences in appearance that would be perceived during the chameleon effect, and to thereby investigate the relationship between translucency and the chameleon effect. The results show that translucency and ∆E are in an inverse relationship, with ∆E = − 52.003 + (1802.608/TP) and r = 0.6804. As translucency increases, ∆E decreases. A decrease in ∆E in turn corresponds to an increase in the chameleon effect. Therefore, high translucency materials will have a high chameleon effect.

## Discussion

Translucency is a physical property between opacity and transparency. Transparency allows light to pass through a material without scattering. This will occur in materials with a uniform refractive index. A translucent material, in contrast, allows only a limited amount of light to pass through its structure, and there is some internalized scattering. A translucent material consists of components with different refractive indices^25^. The refractive index of each component in a material influences the direction of light. The main components of resin composite are resin matrix and inorganic fillers. If both components have similar refractive indices, then there is a little scattering of light and consequently the resin composite will have high translucency^26^. On the other hand, dissimilarity between refractive indices of the resin matrix and the fillers will result low translucency owing to refraction and reflection of light at the matrix-filler interfaces^27^. The number and particle size of the fillers generally have an inverse relationship with the translucency of materials^28^. In this study, the resins of the conventional and flowable resin composite contain the same monomers: Bis-GMA, UDMA, TEGDMA, and Bis-EMA. The refractive indices of these monomers fall in the range of 1.49–1.56^29^. The conventional and flowable resin composites also share the same fillers: non-aggregated silica (refractive index 1.47), zirconia (refractive index 2.15)^30^, and aggregated silica-zirconia clusters. However the flowable resin composite has larger silica particles (75 nm, compared to 20 nm for conventional resin composite) and a smaller total amount of fillers (65% by weight or 55% by volume, compared to 78.5% by weight or 63.3% by volume for conventional resin composite). Even though the size of the silica particles in the flowable resin composite is larger than those in the conventional resin composite, they are still smaller than the wavelength of the curing light. Because of this, the material translucency will be influenced more by the total amount of fillers than by the particle size. The bulk-fill resin composite has all three previously mentioned fillers as well as ytterbium trifluoride, which has high translucency. Its refractive index is 1.53, which is very close to that of both resin monomer (1.49–1.56) and resin-filled enamel (1.52)^29^. Because the refractive index of the ytterbium trifluoride is so close to that of the resin matrix, there is less scattering of curing light and higher penetration into the bulk-fill resin composite.

As previously mentioned, the resin-filled ceramic blocks in this study were thermocycled for an in vivo equivalent of 5 years. The water sorption and solubility of materials are also important properties that affect clinical usage. The nanofilled resin composite materials used in this study have higher water sorption than other microfilled or microhybrid resin composites, but they have lower solubility^31,32^. The surface roughness of these resin composites is not different from other products made with the same types of materials, for example conventional nanofilled, bulk-fill, or flowable resin composites. However conventional nanofilled resin composite has a rougher surface compared with bulk-fill resin composite^33^. This could cause conventional nanofilled resin composite to become more easily discolored.

There are two ways to evaluate the translucency of materials: absolute translucency and relative translucency. Absolute translucency directly measures the transmittance of light, while relative translucency is calculated from the reflectance of the material^19,34^. There are in turn two techniques for measuring the relative translucency: the contrast ratio (CR) and the translucent parameter (TP). The CR compares the reflectance of the material on a black background to the reflectance on a white background. The TP is calculated from the color difference of the material, which is measured in the CIE protocol, on a black versus a white background^20^. This study selected the TP technique, since this technique was developed in relation to human visual perception^34^. This technique more closely resembles the clinical situation.

Within each type of restorative material, when the chroma increased, the translucency increased only insignificantly. These results were in contrast to a previous study^35^. However, even though the differences in translucency of different chromas did not reach statistical significance, it is possible that the chroma might still have some effects on translucency without crossing the threshold for statistical significance in this study.

Surface characteristics of samples also affected on the behavior of light. Surface finishing of restorative materials has a mechanical impact on light scattering and therefore also affects the translucency of the materials^36,37^. This is why the glass slide was applied during polymerization. Controlling the samples in this way produces consistent, uniformly smooth surfaces. Of course the subsurface characteristics of an applied material also influence its translucency. Layered application of resin composite reduces straight-line transmission of light, compared to bulk-fill application^38^. The depth of the ceramic wells was thus made 2 mm in order to allow complete curing of bulk-fill applied resin composites and prevent the junctions that result from layers. The transmission coefficient of light was previously found to decrease as tooth enamel becomes dehydrated. When the enamel is then rehydrated and water again contacts the enamel prism, the transmission coefficient of light rises back^39^. To avoid such dehydration, after the samples in this study were drained from the thermocycling machine, a paper towel was used briefly to remove only excess water before the samples were tested for color.

This study mimics the clinical situation of filling a tooth by using the ceramic instead of a natural tooth. Natural human teeth vary greatly in color, and different parts of one tooth also have different colors. Ceramic blocks were therefore used to eliminate inconsistencies from uncontrollable factors, and this was also a limitation of this study. The ceramic blocks chosen for this study are made of high translucent (HT) zirconia-reinforced lithium disilicate ceramic with an added 10% translucent zirconium oxide. This material is designed to represent natural enamel and dentin. A study in 2014 investigated the optical properties of various ceramics, and high translucent lithium disilicate ceramic was found to have a translucency parameter closest to natural enamel^40^.

When this study investigated the color stability of restorative materials and ceramic that had undergone the equivalent of 5 years of stress, the lightness of both significantly decreased after one year and their yellowness increased after 2 years, while there was no significant change in the redness of the ceramic. Even though there were statistical differences in lightness and yellowness of the ceramic during the first 2 years, the ∆E of the ceramic at the 2 year mark compared with Day 0 was 1.34, which is a difference that only experienced observers can notice and general observers cannot. After 5 years, the ∆E was 2.58, which is a difference that even inexperienced observers are able to detect. Previous research has found that when ∆E is between 2 and 3.5, even inexperienced observer can detect the difference^41–43^. There have been few previous studies on ceramic color stability that did not involve external staining factors such as food and tobacco. A 2017 study investigated the color changes in polished or glazed glass ceramic after it underwent temperature stress equal to only 10 days of normal use. That study found that changes in all three dimensions were only insignificant: decreased lightness and yellowness and increased redness^44^. Those changes were different from the current study, which has a much longer period of thermocycling, where the color changes in the resin composite and glass ionomer cement were more substantial and distinct than changes in the ceramic of the 2017 study. The ∆E of each group was greater than 3.5 at the one year mark, compared with day 0 (data not shown). This indicates that the chameleon effect seen in the current study results from the restorative materials, not the ceramic.

Translucent restorative materials can allow underlying tooth structure to show through, and they can also reflect surrounding tooth structure. Both of these phenomena can result in color changes in restorations. In this study, the flowable, enamel, and body resin composites can be classified as a high translucency material (with no statistical difference in translucency among them) while the dentin and bulk-fill resin composites as well as the glass ionomer cement can be classified as low translucency materials.

The results of this study show considerable variation and complexity. That said, there is a discernable pattern in which the materials that had the best color blending performance can be divided into two groups: materials that produced immediate color blending, i.e. at day 0, and materials that produced delayed color blending, i.e. at a point later than day 0.

Immediate color blending (i.e. a low ∆E value at day 0) was clearly found in eight of the samples: all three shades of the enamel resin composite, shades A1 and A2 of the flowable resin composite, and all three shades of the bulk fill resin composite. Shade A2 of the bulk fill resin composite had the most pronounced immediate color blending (∆E = 1.24) of any material in the study. It is interesting that three of these eight samples with the most distinct immediate color blending are bulk-fill resin composite, even though all three bulk fill shades are in the low translucency group. Since bulk-fill resin composite is the only material in this study with the special added filler ytterbium trifluoride, which has a refractive index equal to that of resin matrix to increase light penetration during curing and thereby facilitate the bulk fill technique. It is possible that the degree of similarity between the refractive indices of each material’s various components might have more influence on that material’s potential for color blending (the chameleon effect) than does the translucency of the material itself. Further studies would be necessary to investigate this.

It is noteworthy that, with the unusual exception of bulk fill resin composite just mentioned, all the other members of this immediate blending group are high translucency materials. In contrast, the low translucency materials other than bulk fill resin composite showed low levels of immediate blending. The translucency of materials applied into cavities thus has a direct effect on immediate color blending.

Twelve materials that produced delayed color blending included all three shades of all three types of conventional resin composite, shades A1 and A3 of flowable resin composite, and the glass ionomer cement. These delayed blending materials improved their ∆E for some period of time after day 0, with the effect continuing for 2 to 5 years. The delayed chameleon effect was significantly distinct in the low translucent materials, except for the bulk-fill resin composite. This might be because the bulk-fill resin has such pronounced immediate blending that the material cannot sustain or improve on that level of color blending afterwards. Some of the materials in this delayed blending group lost this effect in the final years of observation.

Interestingly, the immediate blending group and delayed blending group are not mutually exclusive. There are four crossover materials that produce both good immediate blending and good delayed blending. The four crossover materials are: the three shades of enamel resin composite and the A1 shade of flowable resin composite. Selecting restorative materials from among these four crossovers is likely to provide the most satisfying short- and long-term results. When that is not possible, other materials in the immediate blending group are also good choices.

The chameleon effect of each type and shade of material, investigated here by measurement of the Delta E between ceramic and restorative materials, can be considered for clinical applications involving not only tooth restoration but also ceramic repairment. Resin composite is the material most commonly used to repair broken ceramic restorations, and it forms an excellent chemical bond to glass ceramic. Since this study used glass ceramic to represent natural tooth enamel in a standardized form, the results of this study are certainly pertinent to ceramic repairment in addition to dental fillings.

## Conclusion

The translucency of the materials investigated here had a 68% reverse correlation with their ∆E values, and ∆E is inversely proportional to the chameleon effect. Therefore, the translucency of materials was directly correlated with the chameleon effect. The chameleon effect occurred most frequently and most clearly during the first 3 years. After that the color difference gradually increased.

## Data Availability

All data generated or analysed during this study are included in this published article.
